# Childbirth Experience and Pain Control: Expectation, Satisfaction, and Analgesia Myths

**DOI:** 10.7759/cureus.63082

**Published:** 2024-06-25

**Authors:** Ricardo Rodrigues, Catarina Freitas, Beatriz Gonçalves, Joana Freitas, Jhonny Abreu

**Affiliations:** 1 Anesthesiology, Funchal Central Hospital, Funchal, PRT; 2 Nursing, Hospital Central do Funchal, Funchal, PRT

**Keywords:** labor, neuraxial labor analgesia, myths, pregnancy, maternal satisfaction

## Abstract

Background: In our days, increasing importance has been given to maternal satisfaction as a quality indicator in healthcare services. A positive childbirth experience should meet a woman’s personal and sociocultural beliefs and expectations in every setting. This study aimed to evaluate childbirth experience regarding expectations, satisfaction, and myths around epidural analgesia.

Methodology: A cross-sectional survey designed was carried out in the Obstetric Department of a public hospital in Madeira-Portugal. A well-structured questionnaire was applied to 101 post-partum women covering aspects such as sociodemographic details, childbirth expectations, overall satisfaction, and prevailing myths. IBM SPSS Statistics for Windows, Version 26.0 (IBM Corp., Armonk, NY) was used for data analysis.

Results: From the total of 101 participants, 32 (31%) women belonged to the 31-35 age group. Among the respondents, 58 (57%) had attained a high school diploma. The results showed that there was a positive experience with childbirth; out of the total women, 79 (78%) considered it exceeded their expectations. The majority of pregnant women (93, 92%) received neuraxial analgesia for labor, reporting the experience as good or excellent. The overall satisfaction related to the birth experience was good or excellent for 88 (87%) women. Regarding the myths, education level was significantly associated with the myth *Epidurals often cause permanent back pain* (*P *< 0.05), since women with higher education don’t believe them.

Conclusions: The result of this study proves that, despite the high level of satisfaction with the labor and delivery experience found in our maternity unit, satisfaction remains a complex and dynamic phenomenon.

## Introduction

The experience of labor is an event full of personal, emotional, and sociocultural meaning, which is directly related to the parturient preferences, professionals’ attitudes, and conduct adopted during care, among other factors.

Maternal satisfaction during labor is a multidimensional experience encompassing various aspects of childbirth. As it is influenced by different factors, such as the level of pain management, perceived control over the birthing process, communication, and involvement in decision-making, it can be difficult to evaluate [1]. Several programs have been implemented to provide pregnant women with evidence-based guidance. In line with this, in 2018, the World Health Organization (WHO) released recommendations on intrapartum care aimed at ensuring a positive birthing experience [2]. Personalized and safe assistance based on scientific evidence with an emphasis on women's active participation are important aspects of the quality of care [3].

The perception of sensory events like labor pain is subjective and significantly differs among women. It is influenced by several factors such as psychological, obstetric, and social factors [4]. Ideal labor analgesia should provide effective and safe pain relief, without interfering with the progression of labor and have minimal effects on the fetus and parturient. Furthermore, it should be easy to initiate and maintain, require little monitoring, and be effective throughout the entire birth. Neuraxial analgesia, which includes epidural, subarachnoid, and combined (or sequential) techniques, meets most of these requirements.

Women experience severe pain during labor. Therefore, the pregnancy request is enough for neuraxial analgesia initiation. Nevertheless, the choice of undergoing a neuraxial technique should be a conscious one, so pregnant women should be informed correctly. The myths surrounding neuraxial analgesia may impact women's decision-making processes. They arise from a combination of factors including misinformation, cultural attitudes, fear of medical intervention, limited understanding, and personal experiences. This study aimed to evaluate childbirth experience regarding expectations, satisfaction, and myths around epidural analgesia.

## Materials and methods

A cross-sectional survey designed was carried out in the Obstetric Department of a public hospital in Madeira-Portugal. This hospital attends 1,600 births each year, with a labor analgesia coverage rate of 85%. Several analgesic labor techniques are available, with neuraxial analgesia (including sequential spinal epidural at 64%, epidural at 34%, and subarachnoid block at 1%) being the most prevalent. Less frequently utilized options include remifentanil patient-controlled analgesia (PCA) and nitrous oxide.

A non-probability convenience sampling technique was used. Parturients were recruited over three months in 2023, during the postpartum period, depending on researcher availability.

The eligibility criteria encompassed adults aged 18 years and above, with live newborns, who underwent labor (regardless of delivery method) and did not face any communication difficulties. Exclusion criteria comprised elective cesarean sections, unwillingness to participate, language barriers, and incomplete questionnaire responses. Based on these factors, the researchers determined a total of 101 women.

The study was approved by the Funchal Central Hospital Ethics Committee (approval number 60/2023) and registered in ClinicalTrials.gov (ID S23003697). The informed consent form was required for all women before applying the questionnaire. Completing the questionnaire took an average of 10-15 minutes, it was anonymous and handed to nurse staff before discharge.

A well-structured questionnaire containing 24 closed questions, which consisted of four parts, was developed by the research team. The first part covered information on the sociodemographic characteristics of participants, including age, body mass index (BMI), marital status, education level, nationality, religion, and employment status. The second part evaluated data concerning pregnancy and delivery characteristics, including parity, gestational duration, prenatal care, pregnancy-related risks such as maternal age, complications from previous pregnancies, multiple pregnancies, and pre-existing medical conditions. The third part included data relating to labor analgesia. The final part contained information regarding satisfaction levels and expectations regarding labor and healthcare providers, along with misconceptions surrounding neuraxial analgesia. Satisfaction was evaluated using a five-point Likert scale, ranging from *very poor* to *excellent*.

Statistical analysis was performed using IBM SPSS Statistics for Windows, Version 26.0 (IBM Corp., Armonk, NY). Descriptive analysis was performed for all the variables with frequency and percentages for quantitative variables. The chi-squared test and logistic regression model were used in categorical variables, and the *P*-value < 0.05 was considered statistically significant.

## Results

Sociodemographic characteristics

A total of 101 women who gave birth participated in the study. Table 1 indicates the sociodemographic data of the population studied. According to the data collected and analyzed, most participants (32, 31%) belonged to the 31- to 35-year age group. Portuguese nationals accounted for the majority of the surveyed patients. From the total population of this study, 58 (57%) had graduated from high school and 74 (73%) were employed. The distribution based on religion showed that most individuals followed Christianism (91, 90%).

**Table 1:** Sociodemographic characteristics (n = 101) *n*, number of subjects

Variables	*n* (%)
Age (years)	
<=20	2 (2)
21-25	15 (15)
26-30	23 (23)
31-35	32 (31)
36-45	29 (29)
Nationality	
Portuguese	93 (92)
Others	8 (8)
Marital status	
Single	41 (41)
Married	35 (35)
Cohabiting	24 (24)
Divorced	1 (1)
Education level	
Ninth grade or below	14 (14)
High school	58 (57)
College degree	29 (29)
Employment status	
Employed	74 (73)
Unemployed	27 (27)
Religion	
Catholic	91 (90)
Other	10 (10)

Pregnancy and delivery characteristics

In this study, from the total of 101 women, more than half were primiparas. We also conclude that 42 (86%) women with a previous childbirth had a positive experience. The majority of pregnant women (73, 72%) had gestational age exceeding 37 weeks. Prenatal care was reported in 100 (99%) women.

In the studied population, we found a total of 55 (54%) high-risk pregnant women, with 17 (31%) having chronic conditions. Among these, diabetes was the most prevalent, with 8 (47%) patients in total, followed by hypertension in 6 (35%) patients (Table 2).

**Table 2:** Pregnancy characteristics (n = 101). *n*, number of subjects

Variables	*n * (%)
Parity	
Primiparous	52 (51)
Multiparous	49 (49)
Previous childbirth experience	
Positive	42 (86)
Negative	7 (14)
Gestational age (weeks)	
≥37	73 (72)
<37	28 (28)
Prenatal care	
Yes	100 (99)
Public healthcare	43 (43)
Private healthcare	45 (45)
Both	12 (12)
No	1 (1)
High-risk pregnancy	
Yes	55 (54)
Chronic conditions	17 (31)
Hypertension	6 (35)
Diabetes	8 (47)
Mental illness	2 (12)
Coagulation disturbance	1 (6)
Age >35 years	29 (53)
Others - gemelar, previous miscarriage, previous cesarean section	9 (16)
No	46 (46)

Most pregnant women received neuraxial labor analgesia, accounting for 93 (92%) of the studied population. The main reason for abstaining was a precipitous labor. By the time of doing neuraxial labor analgesia, 70 (75%) women classified pain as severe, according to the visual analog scale (VAS) score. After the technique, 57 (51%) had mild pain and 35 (38%) had moderate pain (Table 3).

**Table 3:** Epidural analgesia (n = 101). *n*, number of subjects

Variables	*n* (%)
Informed consent	
Yes	92 (99)
No	1 (1)
Information about risks/benefits	
Yes	19 (20)
No	74 (80)
Labor epidural analgesia	
Yes	93 (92)
No	8 (8)
Labor pain before epidural (EVA)	
Mild	2 (2)
Moderate	21 (23)
Severe	70 (75)
Labor pain after epidural (EVA)	
Mild	57 (61)
Moderate	35 (38)
Severe	1 (1)
Side effects of epidural analgesia	
Yes	69 (74)
Nausea/vomit	5 (7)
Paresthesia in the lower limbs	12 (17)
Dizziness	2 (3)
Pruritus	8 (13)
Headache	1 (1)
Back pain	1 (1)
Other	2 (3)
More than one	38 (55)
No	24 (26)

All women requesting epidural analgesia should be fully informed of the benefits, side effects, and risks involved, before consenting to the procedure. Informed consent about labor analgesia was requested from 92 (99%) patients, but only 19 (20%) were informed about the risks and benefits of analgesic techniques. The most frequent complaints after neuroaxis analgesia were paresthesia of the lower limbs in 12 (17%) women and pruritus in 8 (13%), often associated (Table 3).

In our survey the most prevalent mode of delivery was spontaneous vaginal birth, constituting 53% of cases in a total of 54 women, followed by cesarean section in 30 women (30%) and dystocic delivery in 17 (17%) cases. The time frame between neuraxial labor analgesia and delivery lasted more than 12 hours in 58 (62%) women.

According to the data collected and analyzed in Table 4, pain from the episiotomy/cesarean was present in 21 (26%) women and back pain after epidural in 13 (16%). They were the most common complaints reported in the postpartum period.

**Table 4:** Postpartum complaints (n = 101). *n*, number of subjects

Variables	*n* (%)
Postpartum complaints	
Yes	81 (80)
Pain (episiotomy/cesarean)	21 (26)
Headache	1 (1)
Back pain	13 (16)
Nipple fissures	2 (2)
Paresthesia in the lower limbs	3 (4)
Other	4 (5)
More than one	37 (46)
No	20 (20)

Satisfaction

Based on the data collected and analyzed in Table 5, the presence of a family member or significant other during labor occurred in 75 (75%) of the cases, with the child's father being the most commonly present. The majority of the participants (89, 88%) had been involved in the decisions about their labor. The quality of maternity facilities was rated as good or excellent by 82 (81%) women, and the quality of care provided by the professionals was rated as good or excellent by 97 (96%). Regarding the birth experience, 79 (78%) women considered it exceeded their expectations.

**Table 5:** Satisfaction scores (n = 101). *n*, number of subjects

Variables	*n* (%)
Labor companionship	
Yes	75 (75)
Father	72 (96)
Significant other	3 (4)
No	26 (25)
Women’s participation in decision-making	
Yes	89 (88)
No	12 (12)
Maternity facilities	
Excellent	20 (20)
Good	62 (61)
Acceptable	18 (18)
Poor	1 (1)
Very poor	0 (0)
Quality of care	
Excellent	66 (65)
Good	31 (31)
Acceptable	4 (4)
Poor	0 (0)
Very poor	0 (0)
Exceeded expectations	
Yes	79 (78)
No	22 (22)
Overall satisfaction	
Excellent	30 (30)
Good	58 (57)
Acceptable	13 (13)
Poor	0 (0)
Very poor	0 (0)
Epidural recommended to family/friend	
Yes	96 (95)
No	5 (5)

The overall satisfaction related to the birth experience was good or excellent for 88 (87%) women. A total of 96 (95%) women would recommend neuraxial analgesia to family members or friends, based on their own experience (actual or previous).

Information

Almost all women in this study had information about labor analgesia (Table 6). Most pregnant women obtained knowledge about labor analgesia from nurses; anesthesiologist was mentioned only by 4 (4%) women. In this study, only one of the patients (1%) didn’t receive information about labor analgesia. Based on the questionnaire assessment, it was a foreign pregnant woman without any prior prenatal care.

**Table 6:** Understanding labor analgesia (n = 101) and epidural myths. *n*, number of subjects

Variables	*n* (%)
Knowledge about labor analgesia	
Yes	100 (99)
Information source	
Family/friends	2 (2)
Internet/books	2 (2)
Nurse	22 (22)
Anesthesiologist	4 (4)
Obstetrician	18 (18)
Family physician	3 (3)
Multiple sources	49 (49)
No	1 (1)
Myths	
Epidural can harm the baby.	2 (2)
I can get an epidural just after 3-4 cm cervix dilatation.	71 (70)
If I have an epidural, I’m more likely to end up needing a C-section.	6 (6)
An epidural makes pushing difficult.	29 (29)
An epidural can leave a woman paralyzed.	48 (48)
Epidurals often cause permanent back pain.	30 (30)
An epidural is not always performed by an anesthesiologist.	7 (7)

Regarding the myths associated with epidural analgesia, 71 (70%) women believe that they only can get an epidural just after 3-4 cm cervix dilatation, and nearly half (48, 48%) women believed that an epidural can leave a woman paralyzed, and 30 (30%) women believed that epidurals often cause permanent back pain (Table 6).

Education level was observed to be significantly associated with the myth *Epidurals often cause permanent back pain* (*P *< 0.05) since women with higher education do not believe them (Table 7).

**Table 7:** Education level and myths. Pearson's chi-squared test; *P* <0.05. X^2 ^(df), chi-square statistic along with its degrees of freedom

Variable	*X*² (df)	P
Epidurals often cause permanent back pain.	6.348	0.042
I can get an epidural just after 3-4 cm cervix dilatation.	3.850	0.146
An epidural can leave a woman paralyzed.	2.259	0.323

A logistic regression was performed to ascertain the effects of age, education level, employment status and parity on the likelihood that *Epidurals often cause permanent back pain* (Table 8). We used the Wald chi-square test to assess the unique contribution of each predictor. The model explained 11.2% of the variance in this myth and correctly classified 72.3% of cases. Increasing educational level was associated with a decreased likelihood of women believing that epidurals often cause permanent back pain.

**Table 8:** Binominal logistic regression model for the myth, Epidurals often cause permanent back pain. Nagelkerke R 0.112; overall percentage 72.3%. Statistically significant for *P *< 0.05 B, coefficient associated with each predictor variable; SE, standard error; Exp, exponentiated coefficient; 95% CI, 95% confidence interval

Variables	B	SE	*P*-value	Exp (*B*)	95% CI
Constant	1.872	1.532	0.222	6.499	
Age	-0.037	0.046	0.420	0.964	0.881-1.054
Educational level			0.093		
High school	-1.31	0.645	0.042	0.270	0.076-0.955
College	-0.622	0.758	0.412	0.537	0.121-2.372
Employment status	-0.626	0.561	0.265	0.535	0.178-1.607
Parity	-0.466	0.545	0.393	0.628	0.216-1.827

## Discussion

This study was conducted in Madeira, Portugal, to evaluate the impact of various factors contributing to maternal satisfaction with the childbirth experience. As the need to enhance healthcare quality continues to grow, ensuring maternal satisfaction remains a pivotal goal for every maternity unit.

The mean maternal age at the birth of their first child in Portugal has been increasing in recent years. According to the National Statistics Institute (INE, 2021), in 2000, the average age of women at the birth of their first child stood at 26.5 years, increasing to 28.1 years in 2010, and further rising to 30.2 years in 2020 [5].

Several factors lead women to become mothers after the age of 35, including individual, family, and societal considerations. Individual factors include independence through education, job security, and financial stability; motivation to have a family; a sense of readiness after achieving personal goals; having a life project; awakening of the biological clock; overcoming or managing chronic health issues such as infertility; and being in a committed relationship. Family-related aspects involve negotiating with the partner who may or may not be prepared for parenthood, the postponement of marriage, and the proximity and support of the main family. In terms of social factors, social acceptance of postponing motherhood, knowledge of high divorce rates and policies that lack maternity support can be considered [6]. These facts are in line with data from this study in which 61 (60%) women in labor were aged over 31 years. The majority of the population studied (87, 86% women) had a high level of education (high school and college degree) and a high employment rate (74, 73%). According to Eurostat, the proportion of births outside marriage has shown an increasing trend in the past decades, in countries like France, Portugal, Bulgaria, Sweden, Slovenia, Estonia, and Spain, reflecting changes in patterns of family formation alongside the more traditional pattern where children were born within marriage [7]. The postponement of marriage is evident in this study, with 65 (65%) women being single/cohabiting.

Many factors influence the outcome of pregnancy starting with the onset of any obstetric/anesthetic complications. Primiparous women are more likely to feel uncertainty and fear related to childbirth than multiparous women [8]. Among multiparous women, previous birth experiences may influence their emotions during the next childbirth. In our research, we found 49 women had a previous childbirth experience, and 42 (86%) classified it as a positive experience. According to the data, satisfaction levels within our maternity unit remain consistent, with 88 (87%) of the 101 participants rating the birth experience as good or excellent. The results reflect the role of the healthcare team and pregnant women in identifying factors that influence the level of satisfaction with the childbirth experience.

Maternal childbirth expectations play an important role in determining a woman's response to her childbirth experience. In our study, the majority of women reported that their expectations were exceeded (79, 78%). Childbirth expectations and women’s beliefs differ significantly from one another, and several factors can influence these varying childbirth expectations (pregnant attitude, social and medical environments, characteristics of the pregnant woman, earlier experience, and social media) [9].

Anesthetists are morally and legally obliged to obtain consent from women before performing regional analgesia in labor. It is also part of good clinical practice. All women requesting epidural analgesia should be fully informed of the benefits, side effects, and risks involved, before consenting to the procedure [10]. Surprisingly, informed consent for the neuraxial approach was not obtained from all women, and of these, only 19 (20%) said they had been informed about the risks and benefits of analgesic techniques. This finding leads us to a reflection on the anesthesiologist’s practice and their role in transmitting information and demystifying erroneous concepts about neuraxial analgesia. Ensuring sufficient availability, comprehension, and utilization of health information is vital, particularly regarding high-risk health behaviors and during vulnerable circumstances, such as laboring pregnant women [11]. It’s imperative to guarantee that informed consent in medical decision-making adheres to legal and ethical standards.

Surprisingly, anesthesiologists are, among the members of the multidisciplinary team, the least mentioned by pregnant women as transmitters of information about analgesic techniques, referred only by 4 (4%) women. Knowing that the vast majority of information is transmitted before hospital admission, anesthesia consultation for pregnant women is not a common practice in our institution, and the anesthesiologist’s first contact with the pregnant woman, in most cases, is in the delivery room, may justify the results obtained in this survey. It would be important to institute not only anesthesia consultation for pregnant women but also public programs to increase health literacy in this area, with time to clarify doubts and in a calmer environment than the delivery room.

Despite the numerous modalities for controlling labor pain, neuraxial techniques (single-shot spinal, standard epidural, and combined spinal epidural) are the most effective techniques to alleviate labor pain. Initiation of neuraxial blockade in laboring patients provides reliable and rapid onset of high-quality pain relief with minimal serious side effects to the mother and fetus [12,13]. Possible side effects of epidural analgesia include headache, soreness in the back, pruritus, transient backache, urinary retention, leg numbness, and hypotension. Complications such as permanent nerve damage are extremely rare [12,14]. This survey showed that out of the total of 101 women, 93 underwent labor analgesia. We found that 70 (75%) pregnant women reported severe pain on the VAS before undergoing the neuraxial technique. After the procedure, 92 (99%) women reported experiencing mild or moderate pain. Despite achieving effective pain management, it is important to acknowledge that satisfaction with childbirth is influenced by various additional factors. Given its significance as a milestone in a woman’s life, childbirth affects her psychologically, physically, and socially.

Expectations can be defined as the pregnant woman’s beliefs about the content, type, and quality of care she will receive. Fulfillment of expectations is one of the most consistent predictors of satisfaction [1]. Satisfaction with the childbirth experience is influenced by multiple factors, including the support system, communication with healthcare providers, birth environment, postpartum support, and various other elements. The level of satisfaction women experience with childbirth serves as a crucial indicator of the quality of maternal care [15,16]. Satisfaction with childbirth has immediate and long-term effects on women's health and the quality of their relationship with their children. Mothers with positive childbirth experiences demonstrate better self-esteem, stronger relationships with their children, and positive expectations of their future childbirths [17,18]. However, satisfaction with childbirth is a complex concept with several dimensions and components, and no consensus exists [18]. According to the present study, the overall satisfaction related to birth experience was positive for most of the women (88, 87%). The factors that contributed to the positive experience in this survey were as follows: the woman's involvement in decision-making, present in 89 (88%) cases; the presence of a family member or significant other during labor, found in 75 (75%) cases; effective pain control in 92 (99%) women; the quality of the maternity unit rated as good or excellent by 82 (81%) women; and the quality of care provided rated as good or excellent by 97 (96%) women surveyed.

Myths sound like ideas to be eradicated from our belief systems, but in medical education research, they provide lessons to be learned and remind us to be cautious in providing and interpreting information [19]. The potential role of women’s individual beliefs perceptions, attitudes, and thoughts should not be ignored regarding how they feel and behave in labor [20]. The myths surrounding labor analgesia can cause confusion and unnecessary concerns for expecting mothers. It's important to know them to better clarify how some of these misconceptions can negatively influence the childbirth experience and subsequent overall satisfaction. Despite the educational background, parity, and younger age of the population being studied, certain myths surrounding neuraxial analgesia endure. Women often hold the belief that epidurals can lead to lasting back pain or paralysis. However, scientific evidence demonstrates that severe complications from an epidural such as paralysis are exceedingly rare. While some women may experience temporary discomfort in the lower back (where the catheter was inserted) for a few hours or days following the epidural, it usually resolves promptly [12,14]. Furthermore, numerous women presume that there’s a limited window for receiving an epidural, an assumption not substantiated by scientific evidence [21], which indicates that an epidural can be administered at any stage of labor solely based on maternal request as an adequate justification.

Limitations

The interpretation of the results of this study should consider possible limitations. We acknowledge that the questionnaire with many questions may have been tiring to complete and may have influenced the marked responses. The difficulty of choosing the ideal moment to deliver the aforementioned questionnaire should be highlighted. We also believe that it may have been challenging for participants to evaluate their experience during their stay at the institution, which may have resulted in less thorough responses. In this type of study, where participants are often asked to recall past events, behaviors, or experiences, inaccuracies in self-reported data may occur. Because it was a time-limited study (three months) conducted in only one hospital, with a limited number of participants, the assessment of prevalence and incidence is limited and cannot be generalized. However, the results obtained regarding satisfaction are consistent with the existing literature.

## Conclusions

This study revealed a high level of satisfaction with the labor and delivery experience among 101 women treated at a Portuguese public hospital. Satisfaction regarding the childbirth experience is complex and unique to each individual. Women may find fulfillment in certain aspects of care while feeling dissatisfied with others, and their views may change over time. Meeting a woman’s expectations is a key factor in ensuring a positive childbirth experience. 

However, greater investment in the qualification of the health education process is recommended, both in the preconception period and the gestational period. The multidisciplinary team must continue to work together with the pregnant woman to define the best intervention plan, always considering her expectations and possible misconceptions about labor. The anesthesiologist must assume a proactive and visible role in the education and implementation of strategies for these women. Improving anesthesiology consultations and organizing multidisciplinary sessions involving obstetricians, anesthesiologists, neonatologists, and nurses for expectant mothers and their support network are recommended strategies to be implemented.
